# A universal cell‐free DNA approach for response prediction to preoperative chemoradiation in rectal cancer

**DOI:** 10.1002/ijc.34392

**Published:** 2022-12-14

**Authors:** Albert Grinshpun, Anatoli Kustanovich, Daniel Neiman, Roni Lehmann‐Werman, Aviad Zick, Karen Meir, Elez Vainer, Roy Z. Granit, Amit Arad, Noa Daskal, Ruth Schwartz, Eli Sapir, Myriam Maoz, Esther Tahover, Joshua Moss, Iddo Z. Ben‐Dov, Tamar Peretz, Ayala Hubert, Ruth Shemer, Yuval Dor

**Affiliations:** ^1^ Sharett Institute of Oncology Hadassah Medical Center Jerusalem Israel; ^2^ The Faculty of Medicine The Hebrew University Jerusalem Israel; ^3^ Department of Developmental Biology and Cancer Research, The Institute for Medical Research Israel‐Canada The Hebrew University‐Hadassah Medical School Jerusalem Israel; ^4^ Department of Pathology Hadassah Medical Center Jerusalem Israel; ^5^ Department of Gastroenterology Hadassah Medical Center Jerusalem Israel; ^6^ Radiotherapy Unit Assuta Medical Center Ashdod Israel; ^7^ Gastrointestinal Oncology Service, Department of Oncology Sha'are Zedek Medical Center Jerusalem Israel; ^8^ Department of Nephrology and Hypertension Hadassah Medical Center Jerusalem Israel

**Keywords:** cell‐free DNA, chemoradiation, methylation, neoadjuvant, rectal cancer

## Abstract

The standard treatment approach for stage II/III rectal cancer is neoadjuvant chemoradiation therapy (nCRT) followed by surgery. In recent years, new treatment approaches have led to higher rates of complete tumor eradication combined with organ‐preservation strategies. However, better tools are still needed to personalize therapy for the individual patient. In this prospective observational study, we analyzed colon‐derived cell‐free (cf)DNA (c‐cfDNA) using a tissue‐specific DNA methylation signature, and its association with therapy outcomes. Analyzing plasma samples (n = 303) collected during nCRT from 37 patients with locally advanced rectal cancer (LARC), we identified colon‐specific methylation markers that discriminated healthy individuals from patients with untreated LARC (area under the curve, 0.81; 95% confidence interval, 0.70‐0.92; *P* < .0001). Baseline c‐cfDNA predicted tumor response, with increased levels linked to larger residual cancer. c‐cfDNA measured after the first week of therapy identified patients with maximal response and complete cancer eradication, who had significantly lower c‐cfDNA compared with those who had residual disease (8.6 vs 57.7 average copies/ml, respectively; *P* = .013). Increased c‐cfDNA after 1 week of therapy was also associated with disease recurrence. Methylation‐based liquid biopsy can predict nCRT outcomes and facilitate patient selection for escalation and de‐escalation strategies.

## Graphical abstract

What's new?

The treatment of locally advanced rectal cancer is a rapidly evolving field, involves multidisciplinary decision‐making, and has no predictive biomarkers for optimal treatment selection. Here, we show that cell‐free DNA from colorectal cells can be identified in patients with rectal cancer, using tissue‐specific methylation markers. Furthermore, cell‐free DNA levels and dynamics are correlated with individuals' outcomes in patients undergoing neoadjuvant chemoradiation for locally advanced rectal cancer.

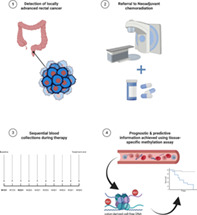

**Abbreviations**
c‐cfDNA: colon‐derived cell‐free DNA
cfDNA: cell‐free DNA
LARC: locally advanced rectal cancer
nCRT: neoadjuvant chemoradiation
pathCR: pathologic complete response
TRG: tumor regression grade

## 1 INTRODUCTION

Cell‐free (cf)DNA released from tumor cells is an emerging substrate for liquid biopsies, used for detection and monitoring of various clinical situations, including non‐invasive prenatal testing, identification of somatic mutations in the plasma of cancer patients, and early detection of graft rejection in organ transplant recipients.^1^ Additional studies suggest that analyses of cfDNA may outperform imaging methods (such as computed tomography), require a lower tumor burden, and prolong the time window for making clinical decisions (eg, detecting molecular relapse months before imaging).^2^

Although somatic mutation detection in cancer and in colorectal cancer in particular is revolutionizing diagnostic oncology, the approach involves significant limitations. We have developed a methylation‐based cfDNA detection assay that can be used for universal detection of tissue‐specific cfDNA without the need for patient‐specific tailoring (tumor agnostic). Moreover, this approach is not affected by clonal hematopoiesis, a recently described and underappreciated confounder.^2^ Previously, we conducted a comparative methylome analysis to identify genomic loci with methylation patterns that are typical of specific cell types and detected these patterns in the plasma of healthy and patient populations. Using bisulfite treatment of cfDNA followed by polymerase chain reaction (PCR) and sequencing, we demonstrated sensitive and specific detection of cfDNA derived from various organs such as the exocrine and endocrine pancreas, liver, heart, breast, and brain.^3^, ^4^, ^5^, ^6^ Our markers enabled us to sensitively detect a response to neoadjuvant chemotherapy in patients with locally advanced breast cancer.^7^ We also have shown that detection of colorectal‐derived (c)‐cfDNA is feasible.^8^

Rectal cancer is a challenging common disease with rising incidence, especially in young adults. The current treatment approach for locally advanced rectal cancer (LARC) is multimodal and includes neoadjuvant chemoradiation (nCRT), followed by surgery, and in some cases by an adjuvant chemotherapy. With modern treatment approaches such as total neoadjuvant therapy (TNT), a significant number of patients is achieving maximal clinical response with complete tumor eradication found on histopathological examination of the resected rectum (complete pathological response, pathCR; or tumor regression grade 0, TRG0). Since pathCR is highly predictive of favorable outcome, significant research efforts are aimed towards the potential omission of surgery in patients achieving complete clinical response, with underlying pathCR. These patients might be offered an organ‐preserving approach with close follow‐up with good outcomes, while avoiding a mutilating surgery and possible permanent stoma placement.^2^, ^9^, ^10^ In the past two decades, numerous trials were aimed to evaluate the optimal approach to predict treatment outcomes, with complete pathological response becoming an acceptable end‐point in clinical trials. Yet, there is no clear consensus on the best approach and the currently available methods are costly, invasive and have limited predictive power.^11^ Currently, more clinical trials are ongoing to further assess the role of various invasive and non‐invasive modalities (such as magnetic resonance imaging, endoscopic biopsies, examinations under anesthesia) in estimation of clinical response in order to personalize therapy (eg, PROSPECT trial [NCT01515787], NCT03426397).

In the current study, we intensively measured c‐cfDNA during nCRT and describe its performance for predicting response to therapy. An early ability of a relatively simple blood test to predict pathCR could affect treatment decisions in both directions: allowing the escalation of therapy for predicted non‐responders and de‐escalation for patients who are expected to experience pathCR, potentially conservative non‐surgical follow‐up.

## 2 MATERIALS AND METHODS

### 2.1 Patients

The study population consisted of patients diagnosed with LARC and treated at the Sharett Institute of Oncology, Hadassah Medical Center, between October 2017 and March 2020. LARC was defined as a node‐positive or T3/T4 biopsy‐proven rectal adenocarcinoma. To exclude metastatic disease, all patients were screened with 18‐FDG‐PET/CT or chest‐abdomen‐pelvis contrast‐enhanced CT. Patients were referred to a long course (5‐6 weeks) of preoperative chemoradiotherapy (54 Gy, including boost) with twice‐daily capecitabine 825 mg/m^2^. Plasma was collected at baseline, and then at the first and last days of each radiation week (up to 5‐6 weeks). All patients underwent surgery including total mesorectal excision, and pathCR was defined as no residual invasive tumor cells in the surgical specimen (modified Ryan Tumor Regression Score 0, TRG0).

Healthy samples were collected from patients before a colonoscopy and who were found to have a normal colon.

### 2.2 Sample preparation and DNA processing

Peripheral blood samples were collected in EDTA‐containing tubes or Streck tubes and centrifuged for 10 min (890 × *g* at 4°C for EDTA‐containing tubes or 1400 × *g* at 23°C for Streck). The supernatant containing plasma was transferred to Eppendorf tubes without disturbance of the cellular layer and centrifuged at 16000 × *g* for 20 min at 4°C. The supernatant then was collected in 2‐ml Maxymum recovery tubes (Axygen) and stored at −80°C. cfDNA was extracted from 2 to 4 ml of plasma using the cfDNA Serum and Plasma Kit (Zymo Research) or a QIAsymphony liquid handling robot (Qiagen). DNA quantity was estimated using the Qubit dsDNA HS assay kit (Thermo Fisher Scientific), and cfDNA was bisulfite converted using the EZ DNA Methylation‐Gold Kit (Zymo Research).

### 2.3 Tissue‐specific methylation markers

Tissue‐specific methylation biomarkers were selected after an analysis of publicly available genome‐wide DNA methylation datasets generated using the Illumina Infinium Human Methylation 450 k BeadChip array (as described previously in).^4^, ^7^ Eight colorectal‐specific markers were used: FGFRL1, Col1, ECH1, cg10900049, cg23460250, cg12462916, cg09094964, and cg15139063 (Figure S1).

At least four CHs (non‐CpGs) were incorporated into each primer in order to ensure that only bisulfite‐converted CpGs were amplified and analyzed. Pooled PCR products were subjected to multiplex NGS using the *NextSeq* 500/550 v2 Reagent Kit (Illumina). Sequenced reads were separated by barcode, and aligned to the target sequence with Bismark, using a computational pipeline (available at https://github.com/Joshmoss11/btseq). Sequence qualities were ignored while minimum alignment score was set to −0.2*(length of sequence). CpGs were considered methylated if “CG” was read and unmethylated if “TG” was read. We then determined the fraction of molecules in which all CpG sites were unmethylated. The fraction obtained was multiplied by the concentration of cfDNA measured in each sample, to obtain the concentration of tissue‐specific cfDNA from each donor. Given that the mass of a haploid human genome is 3.3 pg, the concentration of cfDNA could be converted from units of ng/ml to haploid GE/ml by multiplying by a factor of 303. Sequenced molecules were considered to be unmethylated (for specifically unmethylated markers) if all CpGs on the sequenced DNA fragment were unmethylated. The fraction of DNA derived from the cell type of interest for each marker (for specifically unmethylated markers) was calculated as the fraction of completely unmethylated molecules among all sequenced molecules from the marker region. The coverage and quality statistics of the targeted bisulfite sequencing analysis are for each sample summarized in Table S2.

### 2.4 Circulating KRAS detection in cfDNA

To gain further insight into the biology of colorectal‐derived cfDNA, which may represent radio/chemo‐induced damage to normal intestine, we compared methylation‐derived cfDNA levels to direct plasma mutation analysis. For this study, we measured circulating levels of mutated KRAS molecules, before and during therapy during the same timepoints. cfDNA was extracted as mentioned above. Digital droplet PCR for detection of KRAS mutations was performed using ddPCR KRAS G12/G13 Screening Kit (Bio‐Rad).

### 2.5 Statistical analysis

Correlations and comparisons between patient characteristics, cfDNA, and TRG status were analyzed using the non‐parametric Mann‐Whitney *U* test or Fisher's exact test when appropriate. Survival analyses were evaluated by Kaplan‐Meier plots, and any survival differences between groups were estimated by the log‐rank test. The data obtained were analyzed using R packages and GraphPad Prism 9. Results were considered to be significant at *P* < .05.

## 3 RESULTS

We prospectively recruited 37 patients with LARC receiving standard nCRT and acquired a total of 303 blood samples (median eight samples per patient). In addition, plasma from 29 healthy controls was collected. Patient characteristics are described in detail Table 1 and surgical metrics in Table S1. In brief, at treatment initiation, the average age was 57.4 (range 32.3‐84) years. All patients completed the prescribed treatment, but two patients were lost to follow‐up after surgery. Following nCRT, 18.9% of patients (7/37) experienced an optimal response to therapy, defined as pathCR of the surgical specimen. Nineteen patients received adjuvant chemotherapy with 5‐fluorouracil containing regimen, and one patient was treated with adjuvant pembrolizumab. At a median follow‐up of 25.3 months, nine patients developed disease recurrence, one from the pathCR group, and median time to relapse for the entire population was 20 months. Disease recurrence locations are described in Table 1.

**TABLE 1:** Patient characteristics

	No pathCR (n = 30)	PathCR (n = 7)	*P*
At diagnosis			
Age (y)	58.1	55.9	ns
Range	37.3‐84	44‐67.7	
Sex (M/F)	24/6	5/2	ns
Clinical T stage, n (%)^a^			ns
≤T3	20 (89.9)	5 (83.3)	
T4	3 (13.1)	1 (16.7)	
Clinical N stage, n (%)^b^			ns
Negative	5 (20)	1 (16.7)	
Positive	20 (80)	5 (83.3)	
Mean height from anal verge (cm)	4.9	5.4	ns
Therapy			
Mean radiation dose delivered (Gy)	53.8	53.1	ns
Mean interval between radiotherapy end to surgery (Days)^c^	82	70	ns
After nCRT			
Adjuvant chemotherapy, n (%)	18 (60)	1 (14.2)	ns
Capecitabine	9	0	
FOLFOX/XELOX^d^	9	1	
Post nCRT TRG, n (%)			
TRG0	0	7 (100)	—
TRG1	11 (36.6)		
TRG2	11 (36.6)		
TRG3	8 (26.6)		
Follow‐up, median (months)	22.57	20.82	ns
Disease relapse, n (%)	8 (26.7)	1 (14.3)	ns
Lung, n	4		
Liver, n		1	
Lung + liver, n	2		
Pelvic recurrence, n	2		
Time to recurrence, median (months)	18.1	8.98	

Abbreviation: ns: not significant.

^a^ Data not available for seven patients in non‐pathCR group and one in pathCR group.

^b^ Data not available for five patients in non‐pathCR group and one in pathCR group.

^c^ Data not available for one patients in non‐pathCR group and three in pathCR group.

^d^ FOLFOX: intravenous regimen containing folinic acid, 5‐fluorouracil and oxaliplatin. XELOX: regimen containing oral capecitabine and oxaliplatin infusion.

**FIGURE 1:**
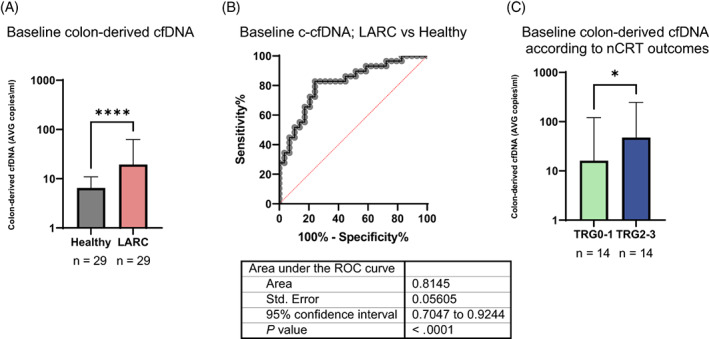
Implications of baseline c‐cfDNA. (A) Median c‐cfDNA levels as measured on diagnosis of patients with LARC, compared with c‐cfDNA measured in individuals immediately before normal colonoscopy. ****Two‐tailed Mann‐Whitney *U* test, *P* < .0001. (B) Receiver operating characteristic (ROC) curve evaluating the discrimination of healthy controls and LARC patients. (C) Pretreatment baseline median c‐cfDNA levels were lower in patients who experienced complete or near‐complete pathologic response to neoadjuvant chemoradiation. *One‐tailed Mann‐Whitney *U* test, *P* = .038

Following application of our methylation assay to plasma from 29 healthy individuals and 29 patients with LARC, we identified a significant difference in median c‐cfDNA levels (Figure 1A, B), with an area under the curve of 0.88 (95% confidence interval, 0.76‐1; *P* = .02). Of interest, the significant difference between healthy controls and patients persisted regardless of treatment response (TRG status).

To further assess the association between TRG and c‐cfDNA, we compared the cumulative level of c‐cfDNA before and during nCRT in patients who reached various TRG scores (Figure 1C). At baseline, patients who experienced complete or near‐complete response to nCRT (TRG0‐1) had lower levels of c‐cfDNA in comparison to patients who experienced a poor response (TRG2‐3) to therapy (n = 14 in each group; *P* = .03, one‐tailed Mann‐Whitney *U* test).

**FIGURE 2:**
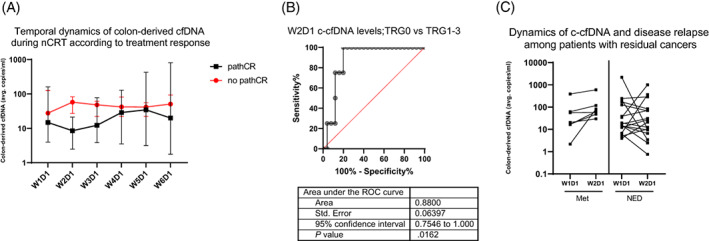
Dynamics of c‐cfDNA during nCRT. (A) Median c‐cfDNA levels as measured during nCRT in patients dichotomized according to treatment response. A significant difference was found at W2D1 (two‐tailed Mann‐Whitney *U*, *P* = .013. (B) Receiver operating characteristic (ROC) curve analysis of the discrimination between patients reaching pathCR and those with residual cancers. (C) Dynamics of c‐cfDNA between baseline and the first day of the second week of therapy were associated with disease recurrence. All patients (100%) who experienced relapse had an increase in c‐cfDNA vs 41% of patients without disease recurrence (two‐tailed Fisher's exact, *P* = .019)

Overall, we observed a trend to elevation of c‐cfDNA during nCRT (Figure 2A). To gain further insights into these dynamics with less noise, we decided to focus on the first measurement in each radiation week (Figure 2B). The first day of each treatment week followed a weekend without radiation or chemotherapy. Dichotomizing patients according to treatment response created two c‐cfDNA dynamics patterns during nCRT (Figure 2A). We found that c‐cfDNA levels on the first day of the second week (W2D1, before nCRT initiation in that specific week) were significantly associated with treatment outcomes. In line with the known decline in cfDNA during treatment response, patients who experienced complete response after nCRT had a median of only 8.6 copies/ml, in comparison with 57.7 copies/ml in patients who still had residual disease (*P* = .013, two‐tailed Mann‐Whitney *U*). Furthermore, c‐cfDNA levels at this timepoint appeared to be predictive of pathCR, with an area under the curve of 0.88 (95% confidence interval, 0.75‐1, *P* = .016) (Figure 2B).

To analyze the correlation between c‐cfDNA and disease‐free survival, we examined the total cfDNA and c‐cfDNA levels at baseline among patients who had disease relapse vs those who did not. We found no correlation, and both groups had similar levels of total cfDNA and c‐cfDNA (*P* > .05). However, when examining patterns of c‐cfDNA dynamics (Figure 2C), we noted that among patients with residual cancer following nCRT (n = 7), all of those who suffered from disease recurrence had increased c‐cfDNA levels from baseline (W1D1) to the beginning of the second week of therapy (W2D1), whereas 7/17 (41%) patients without disease recurrence had an increase in c‐cfDNA levels (proportion comparison, *P* = .019, Fisher's exact). Taken together, these data suggest that a c‐cfDNA increase might hold biologic relevance and represent lack of tumor response to therapy.

**FIGURE 3:**
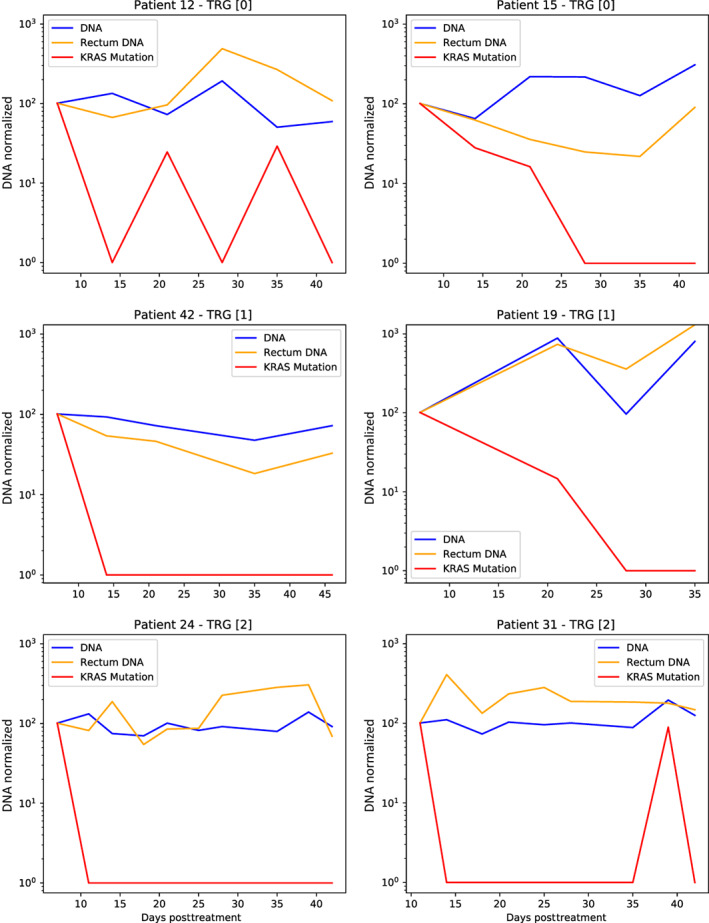
Circulating levels of KRAS during nCRT, in comparison with c‐cfDNA and total DNA in plasma

In our comparison of methylation‐derived cfDNA levels to direct plasma mutation analysis, we measured circulating levels of mutated KRAS molecules, before and during therapy at the same timepoints. At baseline, 16.2% of patients (6/37) had a detectable KRAS mutation in cfDNA, discovered using the digital‐droplet PCR assay. As expected, KRAS levels dropped quickly, while levels of c‐cfDNA continued to be detectable during nCRT, probably representing tissue damage (Figure 3). Moreover, at treatment end or the last measurement, all patients had null levels of circulating KRAS. In addition, KRAS baseline presence, levels, and dynamics were not correlated with TRG or disease‐free survival.

## 4 DISCUSSION

In the present study, we show that methylation‐based detection of tissue‐specific c‐cfDNA allows identification of LARC patients and perhaps their stratification according to their anticipated response to therapy. Furthermore, those who experienced a good response had lower baseline colorectal‐derived cfDNA, and those with maximal response (pathCR, TRG0) could be identified as early as the beginning of the second week of nCRT therapy. Additionally, an increase in c‐cfDNA at the second week of therapy in comparison to baseline levels might hold negative prognostic significance. Similar dynamics have been demonstrated in a small LARC cohort using mutation‐based cfDNA analysis, but those values were not correlated with patient outcomes.^12^ Thus, we believe that this tool can potentially be used to guide therapy in patients with LARC, with clear clinical ramifications, such as directing those with potential non‐response to TNT to increase chances for better TRG.

Of note, our findings are in line with in vitro and in vivo models showing the typical delayed release of cfDNA following tumor irradiation and the known association of cfDNA decrease with tumor eradication.^13^, ^14^ In our cohort, we observed a transient decrease in c‐cfDNA but only in those experiencing maximal response and who later had increased c‐cfDNA levels.

Mutation‐based analysis showed rapid clearance of KRAS from the circulation, consistent with tumor regression. Taken together, we believe that using tissue‐specific markers, we can measure at each timepoint a cumulative amount of tumor‐ and normal‐tissue‐derived cfDNA molecules. Cancer therapy (ie, nCRT) leads to two parallel processes: tumor damage and healthy tissue damage. We assume that most of the c‐cfDNA at baseline was tumor‐derived (similar to KRAS levels), but that with time, it decreased along with a parallel increase in cfDNA from damaged normal intestine (regardless of TRG). Similarly, we have recently shown that collateral tissue damage from metastases leads to elevated cfDNA levels from the damaged organ.^15^ We hypothesize that the beginning of the second week is the accurate timepoint that enables identification of patients whose tumor will respond significantly to a week of nCRT, before the masking effects of normal c‐cfDNA. Likewise, we have observed that increased c‐cfDNA at this timepoint is potentially detrimental, emphasizing that c‐cfDNA at the W2D1 timepoint probably represents the tumor. Of interest, our findings are consistent with other reports showing that short‐term dynamics are predictive of therapy response in colorectal cancer and other tumor types with various treatment modalities.^16^, ^17^

Our method of universal detection has several advantages in comparison with other approaches, such as mutation‐based cfDNA measurements. First, in our cohort, all patients had a baseline signal, in contrast to other studies using mutation panels and showing limited detection of cfDNA at baseline (as low as 21%^18^). We note that Zhao and colleagues reported that all patients who experienced pathCR had no baseline cfDNA by mutation detection.^19^ Second, methylation‐based methods do not require a priori knowledge of an individual's mutations and therefore save time and resources.^12^, ^18^ Approaches using pre‐determined panels might fail to capture up to 60% of patients,^20^ and whole‐exome‐based tools are costly and require more resources. Third, methylation‐based methods are insensitive to potential disturbances inflicted by clonal hematopoiesis of indeterminate potential. Fourth, because this approach detects colorectal‐derived cfDNA, it can provide information about normal tissue dynamics in response to nCRT.

Our work has several limitations, mainly the small sample size and relatively short follow‐up period. Also, in contrast to other studies, our markers showed significance when they were used at more than one timepoint, probably because of their ability to detect normal tissue‐derived cfDNA and not strictly cancer‐related alterations. Nevertheless, we believe that the results are promising and warrant larger clinical trials. Given the advantages of methylation‐based assays, further studies to dissect the epigenetic differences between normal and cancerous cfDNA from the same organ are of great importance. Notably, a recent progress in this field was done by Sadeh et al., analyzing cfDNA active chromatin modifications in order to get insights into cancer cells' transcriptomics.^21^

In conclusion, we have shown that methylation‐based cfDNA analysis predicts outcomes of chemoradiation and may facilitate future studies to deepen our understanding of real‐life tumor biology under radiotherapy treatments. Therapy monitoring using cfDNA is feasible and may offer clinicians necessary information to personalize therapy and achieve better outcomes.

## AUTHOR CONTRIBUTIONS

Albert Grinshpun: Conceptualization, analysis, funding acquisition, investigation, methodology, writing‐original draft, review & editing, Anatoli Kustanovich: analysis, investigation, Daniel Neiman: analysis, investigation, methodology, Roni Lehmann‐Werman: investigation, methodology, Aviad Zick: analysis, investigation, writing‐review & editing, Karen Meir: investigation, Elez Vainer: investigation, Roy Z Granit: analysis, investigation, Amit Arad: investigation, data curation, project administration, Noa Daskal: investigation, data curation, project administration, Ruth Schwartz: investigation, data curation, Eli Sapir: investigation, conceptualization, Myriam Maoz: investigation, methodology, Esther Tahover: investigation, Josh Moss: analysis, investigation, methodology, validation, Iddo Z Ben‐Dov: analysis, visualization, Tamar Peretz: resources, funding acquisition, Ayala Hubert: resources, supervision, Ruth Shemer: conceptualization, investigation, methodology, validation, Yuval Dor: conceptualization, analysis, funding acquisition, supervision, investigation, methodology, writing‐review & editing. The work reported in the paper has been performed by the authors, unless clearly specified in the text.

## FUNDING INFORMATION

The study was supported by the Israeli Ministry of Science and Technology precision medicine grant 2018, and the Israel Science Foundation (ISF) physician‐scientist grant (3020/20).

## CONFLICT OF INTEREST

Ruth Shemer and Yuval Dor filed a patent (2019, application number 62/828587) on cell‐free DNA analysis technology. The other authors do not declare a conflict of interest.

## ETHICS STATEMENT

Our study was conducted in accordance with the principles of the Declaration of Helsinki and was approved by the Hadassad Institutional Ethics Committee (protocols 346‐12, 91‐21). All patients provided written informed consent.

## Supporting information


**APPENDIX S1.** Supporting Information

**TABLE S2.** Coverage and quality statistics of the targeted bisulfite sequencing analysis.

## Data Availability

The data that support the findings of our study are available from the corresponding author upon reasonable request.
