# Supplementary material for: A universal cell‐free DNA approach for response prediction to preoperative chemoradiation in rectal cancer
Source: Int J Cancer. 2022 Dec 14;152(7):1444–51. doi: 10.1002/ijc.34392 (PMC10108011; doi:10.1002/ijc.34392)
Supplement: Supplementary file 1 — APPENDIX S1. Supporting Information [file IJC-152-1444-s002.pdf]

Supplementary Material

A universal cell-free DNA approach for response prediction to preoperative chemoradiation in rectal cancer

Albert Grinshpun, Anatoli Kustanovich, Daniel Neiman, Roni Lehmann-Werman, Aviad Zick, Karen Meir, Elez Vainer, Roy Z Granit, Amit Arad, Noa Daskal, Ruth Schwartz, Eli Sapir, Myriam Maoz, Esther Tahover, Josh Moss, Iddo Z Ben-Dov, Tamar Peretz, Ayala Hubert, Ruth Shemer, Yuval Dor

Content:

1. **Supplementary Table 1**: Additional clinicopathological characteristics of study population
2. **Supplementary Figure 1**: Tissue panel of eight colon-specific methylation markers.
3. **Supplementary Table 2**: Coverage and quality statistics of the targeted bisulfite sequencing analysis. Due to file size, available as separate excel sheet.

Supplementary Table 1. Additional clinicopathological characteristics of study population

	No pathCR (n = 30)	PathCR (n = 7)	*P*
Total mesorectal excision (%)	100%	100%	*ns*
Low anterior resection/ Abdominoperineal resection/unknown (%)	67%/30%/3%	56%/0/44%	*ns*
Distal surgical margins involvement[#]	14%		
Circumferential margins involvement[#]	10%		
Lymph node harvest (mean)[$]	19.6	19	*ns*
Lymphovascular \ perineural invasion (%)[$]	27%	0%	$P < 0.05$
Mismatch repair deficiency (%)[^]	9.5%		

ns – not significant

[#] Data not available for 1 patient

[$] Data not available for 2 patients in non-pathCR group and 3 in pathCR group

^ Data available for 21 patients (70%)

Supplementary Figure 1: Tissue panel of eight colon-specific methylation markers.

The percentage of molecules with colon-specific methylation in genomic DNA was obtained from different cell types and tissues. In these experiments, we determined the fraction of molecules that were largely unmethylated across amplified CpG sites.

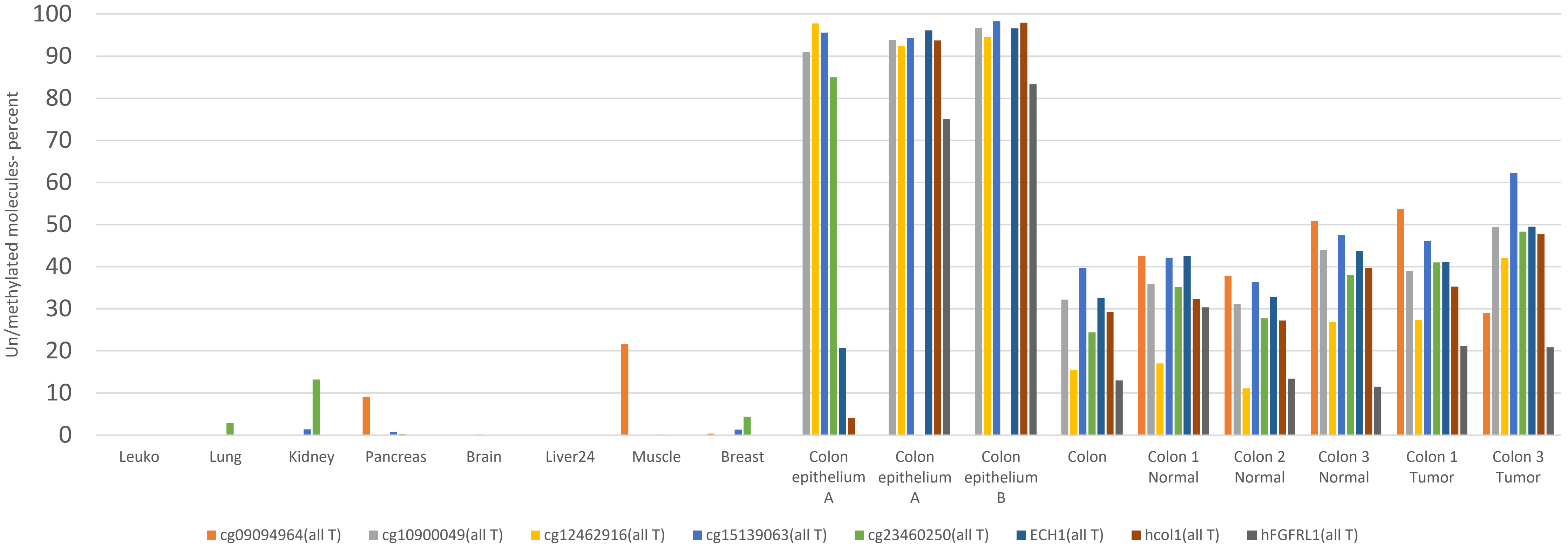

Un/methylated molecules- percent
100
90
80
70
60
50
40
30
20
10
0
Leuko
Lung
Kidney
Pancreas
Brain
Liver24
Muscle
Breast
Colon epithelium A
Colon epithelium A
Colon epithelium B
Colon
Colon 1 Normal
Colon 2 Normal
Colon 3 Normal
Colon 1 Tumor
Colon 3 Tumor
cg09094964(all T)
cg10900049(all T)
cg12462916(all T)
cg15139063(all T)
cg23460250(all T)
ECH1(all T)
hcol1(all T)
hFGFRL1(all T)
